# Caffeine Intake May Modulate Inflammation Markers in Trained Rats

**DOI:** 10.3390/nu6041678

**Published:** 2014-04-21

**Authors:** Rômulo Pillon Barcelos, Mauren Assis Souza, Guilherme Pires Amaral, Silvio Terra Stefanello, Guilherme Bresciani, Michele Rechia Fighera, Félix Alexandre Antunes Soares, Nilda de Vargas Barbosa

**Affiliations:** 1 Departamento de Química, Centro de Ciências Naturais e Exatas (CCNE), Universidade Federal de Santa Maria (UFSM), 97105-900 Santa Maria, Brazil; E-Mails: romulo1604@hotmail.com (R.P.B.); maurensouza@gmail.com (M.A.S.); guipiresa@yahoo.com.br (G.P.A.); silviotstefanello@hotmail.com (S.T.S.); guilhermebresciani@gmail.com (G.B.); nvbarbosa@yahoo.com.br (N.V.B.); 2 Laboratório de Bioquímica do Exercício (BioEx), Centro de Educação Física e Desportos, Universidade Federal de Santa Maria (UFSM), 97105-900 Santa Maria, Brazil; E-Mail: mrfighera@yahoo.com.br (M.R.F.); 3 Facultad de Ciencias de la Salud, Universidad Autónoma de Chile, 4810101, Temuco, Chile;; 4 Departamento de Neuropsiquiatria, Centro de Ciências da Saúde (CCS), Universidade Federal de Santa Maria (UFSM), 97105-900 Santa Maria, Brazil

**Keywords:** caffeine, exercise training, mitochondria, inflammation, myeloperoxidase

## Abstract

Caffeine is presented in many commercial products and has been proven to induce ergogenic effects in exercise, mainly related to redox status homeostasis, inflammation and oxidative stress-related adaptation mechanisms. However, most studies have mainly focused on muscle adaptations, and the role of caffeine in different tissues during exercise training has not been fully described. The aim of this study was therefore, to analyze the effects of chronic caffeine intake and exercise training on liver mitochondria functioning and plasma inflammation markers. Rats were divided into control, control/caffeine, exercise, and exercise/caffeine groups. Exercise groups underwent four weeks of swimming training and caffeine groups were supplemented with 6 mg/kg/day. Liver mitochondrial swelling and complex I activity, and plasma myeloperoxidase (MPO) and acetylcholinesterase (AChE) activities were measured. An anti-inflammatory effect of exercise was evidenced by reduced plasma MPO activity. Additionally, caffeine intake alone and combined with exercise decreased the plasma AChE and MPO activities. The *per se* anti-inflammatory effect of caffeine intake should be highlighted considering its widespread use as an ergogenic aid. Therefore, caffeine seems to interfere on exercise-induced adaptations and could also be used in different exercise-related health treatments.

## 1. Introduction

Aerobic physical training demands great amounts of energy turnover, which is mainly promoted by increased oxygen consumption. In this sense, it is well known that exercise induces several metabolic changes, which can disrupt the mitochondrial functioning in different ways [1]. Among them the oxygen uptake rate during exercise training is considered an important factor to the mitochondrial excessive reactive oxygen species production (ROS) [2]. In this context, mitochondrial dysfunction seems to be closely related to oxidative damage caused by exercise in different tissues [3].

Considering the complexity of exercise-induced cell damage, more comprehensive strategies to understand the associated mechanisms are of interest. In this line, mitochondria are the major site of cellular ROS production while at the same time are also ROS targets [4,5], indicating that mitochondrial dysfunction play a key role in exercise performance [6]. Of note, we have recently described a positive antioxidant modulation of liver mitochondria to exercise training [6]. As such, mitochondria could also bring to light relevant information on exercise mediated-cell antioxidant adaptation.

In animal models, chronic exercise has been attributed a key role in tissue homeostasis, associated with both increased antioxidant defenses and aerobic metabolism [7] and decreased liver inflammation [8], as well as the stimulation of tissue turnover [9,10]. Additionally, epidemiological data and human intervention studies have confirmed the potential benefits of low-to-moderate intensity chronic exercise on muscle health [11,12]. However, studies considering exercise-related adaptations on the liver are still scarce [13].

Caffeine is a xanthine alkaloid compound presented in many commercial beverages and medicines, and its concomitant use with regular exercise may influence the physiological response to effort [14]. Ergogenic effects of caffeine are mainly on central and peripheral mechanisms [15,16,17], but there is a lack of information concerning its chronic effects. A few studies have suggested that chronic caffeine intake decreases inflammatory injury and chronic inflammation in the liver and brain [8,18,19]. These studies have attributed this protective effect to the antioxidant effects of chronic caffeine intake and decreased activation of resident macrophages (Kupfer cells) and microglia. Moreover, chronic caffeine intake decreases the expression of inflammatory cytokines in blood monocytes and resident macrophages, indicating it may chronically decrease local inflammation [19,20]. Besides, in a mouse model of liver injury, chronic caffeine intake decreased the expression of the pro-inflammatory cytokines TNF-α, IL-6 and IL-1β [19].

However, it is still unknown whether caffeine acts as an energetic buffer and/or trigger of peripheral mechanisms of antioxidant and inflammation modulation. Although there is evidence suggesting beneficial effects of chronic caffeine supplementation on oxidative stress markers, the mechanisms by which these adaptations occur are still to be clarified. In addition, the interaction between exercise and caffeine in the liver is poorly described in the literature, despite the remarkable role of this organ on energy turnover during exercise. Therefore, the aim of this study was to investigate the effect of chronic caffeine intake in liver mitochondria and plasma markers of oxidative metabolism and inflammation in trained rats.

## 2. Materials and Methods

### 2.1. Animals and Reagents

Male Wistar rats (180–250 g) were obtained from our own breeding colony and kept in plastic boxes containing a maximum of five animals per cage under controlled environment conditions (12:12 h light-dark cycle, with onset of light phase at 7:00, 25 ± 1 °C, 55% relative humidity) with food (Guabi, Santa Maria, Brazil) and water *ad libitum*. All experiments were conducted in accordance with national and international legislation (Brazilian College of Animal Experimentation (COBEA) and the U.S. Public Health Service’s Policy on Humane Care and Use of Laboratory Animals-PHS Policy) and with the approval of the Ethics Committee for Animal Research of the Universidade Federal de Santa Maria (UFSM). Assay reagents were purchased from Sigma (St. Louis, MO, USA). The other chemicals were of analytical grade and obtained from standard commercial suppliers.

### 2.2. Training Protocol

For exercise training, animals were weighed (270–340 g) and randomly assigned to the following groups: control, control-caffeine, exercise, and exercise-caffeine. The training consisted of four weeks of swimming, 50 min per day and five sessions per week. The tank used in this study was 80 cm in length, 50 cm in width, and 90 cm in depth, and the swimming training was performed in water temperature of 31 ± 1 °C (70 cm depth) between 10:00 and 12:00 a.m. The exercise groups performed the swimming training with a 5% body weight overload attached to the back to improve endurance [21]. The control groups were placed in a separate but similar tank with shallow water (5 cm) at the same temperature for 30 min, five days a week without the back overload. Caffeine administration was performed daily by intragastric gavage at a dose of 6 mg/kg (in saline) throughout the training protocol [22]. Control groups received saline. Animals were sacrificed 24 h after the last training bout to avoid possible exercise bias.

### 2.3. Liver Mitochondrial Parameters

#### 2.3.1. Mitochondria Isolation

The liver mitochondria were isolated at 4 °C by differential centrifugation [23] with some modifications. The sample of the liver was rapidly removed and immersed in ice-cold “isolation buffer I” at 4 °C (100 mM sucrose, 10 mM EDTA, 100 mM Tris-HCl, 46 mM KCl, at pH 7.4). The tissue was then homogenized and the resulting suspension was centrifuged for 5 min at 2000× *g* in a Hitachi CR21E centrifuge (Koki, Tokyo, Japan). After centrifugation, the supernatant was recentrifuged for 20 min at 12,000× *g*. The pellet was gently resuspended in “isolation buffer II” (100 mM sucrose, 10 mM EDTA, 100 mM Tris-HCl, 46 mM KCl, and 0.5% fatty-acid free bovine serum albumin (BSA) free of fatty acids, at pH 7.4) and recentrifuged at 12,000× *g* for 10 min. The supernatant was decanted, and the final pellet was gently washed and resuspended in “isolation buffer III” (270 mM mannitol, 70 mM sucrose, 20 mM Tris-HCl at pH 7.4).

#### 2.3.2. Measurement of Mitochondrial Transmembrane Electrical Potential (ΔΨ*m*)

The mitochondrial ΔΨ*m* determination was estimated by fluorescence changes in safranine-O assayed according to Akerman and Wikstron (1976) [24]. The fluorescence analysis was performed at 495 nm for excitation and 586 nm for emission, with slit widths of 5 nm. The ΔΨ*m* was presented as arbitrary fluorescence units per second (AFU/s).

#### 2.3.3. Estimation of Mitochondrial ROS Production

The mitochondrial generation of ROS was determined spectrofluorimetrically using the membrane permeable fluorescent dye H2-DCFDA [25]. The fluorescence analysis was performed at 488 nm for excitation and 525 nm for emission, with slit widths of 5 nm.

#### 2.3.4. Mitochondrial Swelling

Measurement of mitochondrial swelling was performed using a RF-5301 Shimadzu espectrofluorometer at 600 nm and slit 1.5 nm for excitation and emission. The mitochondria (0.1 mg protein/mL) were incubated in the presence of 100 µM Ca^2+^ [26]. Data for mitochondrial swelling was expressed as arbitrary absorbance units per second (AAU/s).

#### 2.3.5. Mitochondrial Complex I Assay

The activity of complex I (NADH dehydrogenase) was measured by following the oxidation of NADH [27,28]. Absorbance at 600 nm was monitored for 2 min to follow the rate of oxidation of NADH, and the activity was determined using an extinction coefficient of 6.22 mM^−1^·cm^−1^. After thawing, the mitochondria were found to be completely permeable to NADH. Results are expressed as % of control.

### 2.4. Myeloperoxidase (MPO) Activity

The plasma activity of the pro-inflammatory MPO enzyme was measured spectrophotometrically by a modified peroxidase-coupled assay system involving phenol, 4-aminoantipyrine (AAP) and H_2_O_2_ as previously described [29]. The results were expressed in micromol of quinoneimine produced at 30 min.

### 2.5. Acetylcholinesterase (AChE) Activity

The AChE activity was estimated in plasma by the Ellman method [30], using acetylthiocholine iodide (ATC) as substrate and etopropazine as butyrylcholinesterase (BChE) inhibitor [31]. Data were expressed in µmol of hydrolyzed ATC/min/mL.

### 2.6. Protein Determination

The protein content was determined as described previously [32] using bovine serum albumin (BSA) as standard.

### 2.7. Statistical Analysis

Data are expressed as means ± SEM. Statistical analysis was performed using two-way analysis of (ANOVA), followed by Student-Newman-Keuls test when appropriate or two-way analysis of variance to determine possible interactions. Values of *p* < 0.05 were considered significant.

## 3. Results

### 3.1. Liver Mitochondrial Parameters

#### 3.1.1. Liver Mitochondria Oxygen Metabolism

**Figure 1:**
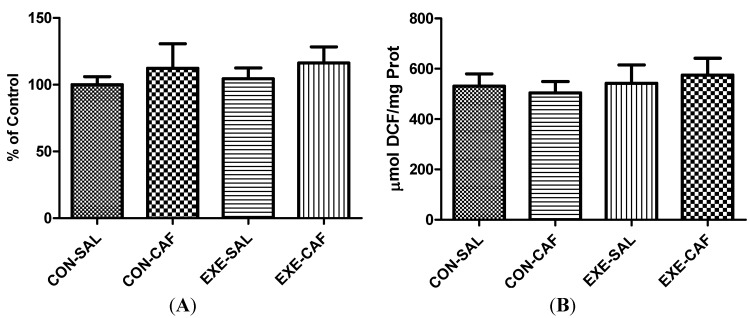
the effects of chronic caffeine intake and exercise training on (**A**) mitochondrial complex I activity; and (**B**) ROS production. Means without a common letter differ significantly (*p* < 0.05). CON: control; CON-CAF: control-caffeine; EXE: exercise; EXE-CAF: exercise-caffeine.

Figure 1 depicts the data regarding oxygen metabolism on liver mitochondria. In this sense, no effect of exercise training or caffeine, nor the combination of both, have affected the activity of the complex I (1A). In same way, no significant differences were found between the groups on mitochondrial ROS production (1B).

#### 3.1.2. Liver Mitochondrial Function

**Figure 2:**
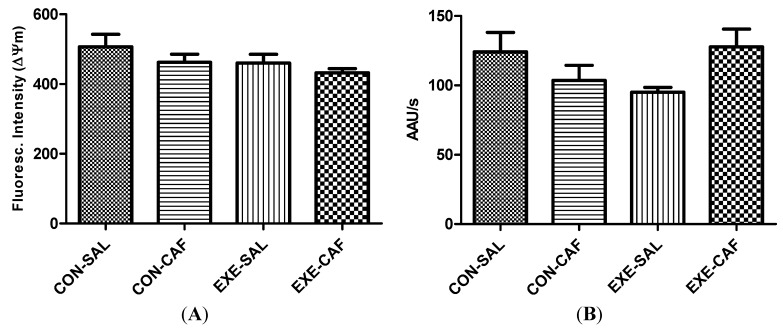
the effects of chronic caffeine intake and exercise training on (**A**) mitochondrial membrane potential; and (**B**) swelling. Means without a common letter differ significantly (*p* < 0.05). CON-SAL: control; CON-CAF: control-caffeine; EXE-SAL: exercise; EXE-CAF: exercise-caffeine.

No effect of the exercise training, caffeine and the combination of both were found on mitochondria functioning parameters. The Figure 2 depicts the data obtained for mitochondria swelling and membrane potential. Exercise, caffeine, and/or control conditions did not affect mitochondrial ΔΨ*m* (2A) and mitochondrial swelling (2B) in the liver.

### 3.2. Inflammation Markers

#### 3.2.1. Myeloperoxidase Activity

**Figure 3:**
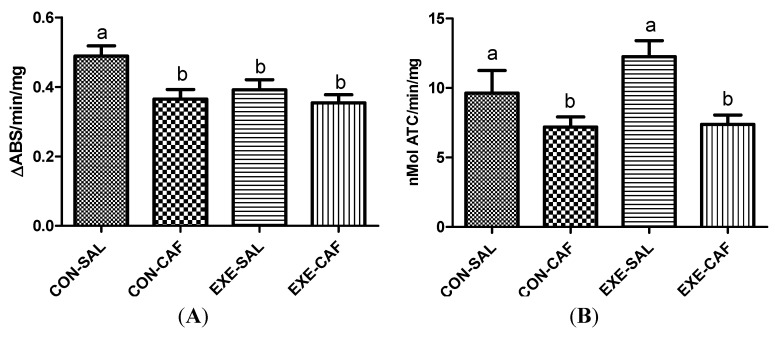
the effects of chronic caffeine intake and exercise training on plasma (**A**) myeloperoxidase; and (**B**) acetylcholinesterase activities. Means without a common letter differ significantly (*p* < 0.05). CON-SAL: control; CON-CAF: control-caffeine; EXE-SAL: exercise; EXE-CAF: exercise-caffeine.

Trained rats exhibited decreased MPO activities when compared with control animals, and caffeine intake did not modify this response when compared to the trained rats (Figure 3A, *p* < 0.05). However, caffeine alone decreased MPO activity when compared to the control rats. The two-way ANOVA did not detect interactions between exercise training and caffeine intake.

#### 3.2.2. Acetylcholinesterase Activity

No differences in AChE activities were observed between control and trained rats. However, caffeine intake decreased AChE activities in both control and exercised rats (Figure 3B, *p* < 0.05). Again, no interaction between exercise and caffeine intake was found by two-way ANOVA.

## 4. Discussion

In this study, we demonstrate a systemic anti-inflammatory role of exercise training evidenced by the reduced MPO activity in plasma. Additionally, we also showed that chronic caffeine alone might modulate systemic inflammatory status herein measured through both MPO and AChE activities. Moreover, chronic caffeine intake has also decreased the AChE activity of trained rats. Despite no changes on mitochondrial function and metabolism, caffeine chronic intake may modulate systemic inflammatory markers combined or not with exercise training.

In a previous study, we have demonstrated the training adaptations of this swimming protocol on specific exercise-related markers [7]. Additionally, this training protocol has also induced liver antioxidant modulation, whereas chronic caffeine intake blunted these adaptations [7]. These were relevant findings due to the massive intake of supplementation commercial products with caffeine used by athletes during training or competitions [14]. Our previous study demonstrated that caffeine acted like an “energy spear” mechanism in the liver, with supplemented rats presenting the same exercise capacity with less metabolic demand in the liver. However, if this mechanism was related to mitochondria energy buffering or to a differential inflammatory modulation was still to be elucidated. Therefore, in this study we investigated whether caffeine could modulate the exercise-related mitochondrial oxidative metabolism and function, and systemic inflammatory markers.

It is hypothesized that aerobic exercise increases ROS production causing oxidative stress and mitochondrial dysfunction [33]. However, in our study, no increase was detected on liver mitochondrial ROS production or any of the other mitochondrial parameters measured among exercised animals. This lack of changes in the liver might also be a consequence of increases observed on the antioxidant defense system, including GPx and SOD activities [7]. These results are in accordance with Sun *et al.* [33] who found no alterations in liver mitochondrial ROS production following exercise training. Apparently, mitochondrial energy turnover and ROS production are directly related to the most active organs during exercise, such as the muscle [34]. We have also previously seen liver mitochondria adaptations on membrane potential and swelling [6], which were not found in this study. A possible explanation for this discrepancy relies on the training intensity and duration, which were both lower in this study. It has been long stated that training duration and intensity are highly related to exercise oxidative metabolism modulation [35].

On the other side, clinical studies and other experimental sets have demonstrated that MPO activity, a marker of neutrophil infiltration, is associated with exercise-induced tissue damage, including muscle, liver, and heart [36,37]. We observed a reduction in plasma MPO activity among trained rats, likely due to the chronic stimulus and mild tissue damage elicited by this swimming protocol. In agreement with our findings, previous studies have shown that exercise training may increase the efficiency of immune functioning and decrease serum levels of inﬂammation markers [10,38,39]. Interestingly, caffeine alone reduced the MPO activity in control rats, demonstrating a *per se* anti-inflammatory role. In this sense, the anti-inflammatory role of caffeine in different tissues of rats has been previously described [8,40].

The hydrolytic enzyme AChE, which is anchored to the membranes of erythrocytes, platelets, leukocytes, and endothelial cells, continuously regulates acetylcholine (ACh) levels [41,42]. ACh has anti-inflammatory functions and suppresses the production of pro-inflammatory cytokines [43,44,45,46]. Thus, ACh levels are reduced when AChE activity is increased, leading to a reduction on the anti-inflammatory actions exerted by ACh [47]. Reduced plasma AChE activity indirectly reduces local and systemic inflammatory events due to the absence of negative feedback control exerted by ACh [47]. In our study, caffeine was able to reduce plasma AChE activity in both control and trained rats, indicating a possible anti-inflammatory role. Accordingly, recent studies have demonstrated that the use of AChE inhibitors suppress systemic inﬂammation and enhance the survival of animals exposed to lipopolysaccharides [48,49] or infection [50]. Regarding exercise, it seems that inflammatory responses rely mainly on duration and intensity [51], which is important considering inflammation blunts exercise performance as seen in studies with different chronic diseases [52,53,54]. Additionally, similarly to the MPO data, the AChE *per se* suppression on control rats is also a remarkable finding to be highlighted. Finally, these data suggest that caffeine could be used in combination with training protocols as a firstline health promotion nutrient. 

## 5. Conclusions

In this study, we demonstrated that exercise training presents anti-inflammatory effects herein evidenced by decreased and MPO activity. Moreover, we have also found a *per se* anti-inflammatory effect of caffeine intake through reduction on both MPO and AChE activities on control animals. These are interesting findings since caffeine has long been used as an antioxidant molecule, in spite of the anti-inflammatory role it may exert. These adaptations are linked to an increased exercise performance as seen in our previous study, which corroborates previous data. In this sense, in this exercise training protocol caffeine is not acting directly on the hepatic oxygen metabolism to induce higher exercise capacity as we have not found liver mitochondria to be affected by caffeine intake or training. Apparently, in this swimming protocol the exercise capacity is related to increased antioxidant (as seen in our previous study) and inflammatory modulation. Future studies are needed to clarify the metabolic pathways related to both antioxidant and anti-inflammatory adaptations elicited by caffeine intake and exercise training.
